# Supplementary material for: Manufacturing of Complex Silicon–Carbon Structures: Exploring SixCy Materials
Source: Materials (Basel). 2022 May 12;15(10):3475. doi: 10.3390/ma15103475 (PMC9146070; doi:10.3390/ma15103475)
Supplement: Supplementary file 1 [file materials-15-03475-s001.zip › materials-1715419-supplementary.pdf]

Supporting information

Manufacturing of Complex Silicon–Carbon Structures: Exploring SixCy Materials

Skyler Oglesby[1], Sergei A Ivanov[2], Alejandra Londoño-Calderon[2], Douglas Pete[3], Michael Thompson Pettes[2], Andrew Crandall Jones[2] and Sakineh Chabi[1*]

[1]Department of Mechanical Engineering, University of New Mexico, Albuquerque, NM 87131, USA

[2] Materials Physics and Application Division, Los Alamos National Laboratory, Los Alamos, NM 87545, USA

[3] Sandia National Laboratories, Albuquerque, NM 87123, USA

* Email: schabi@unm.edu

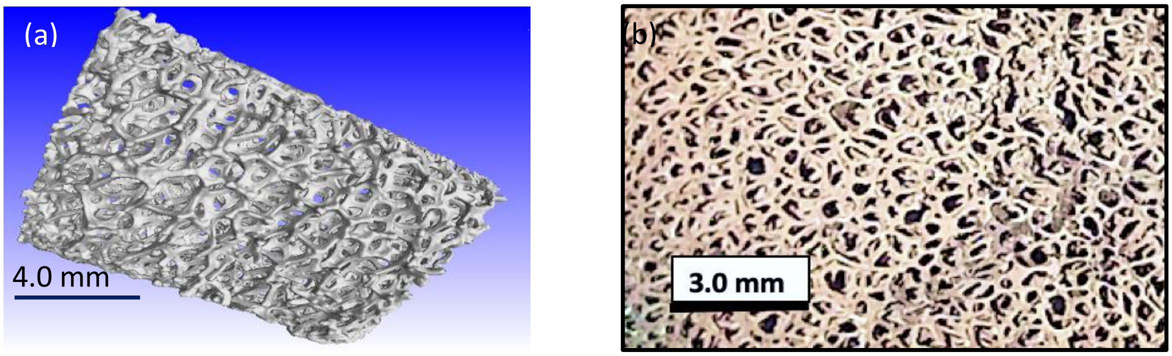

Figure S1. (a) SEM images from the graphene foam precursor, and (b) photograph from the produced Si/C foam.

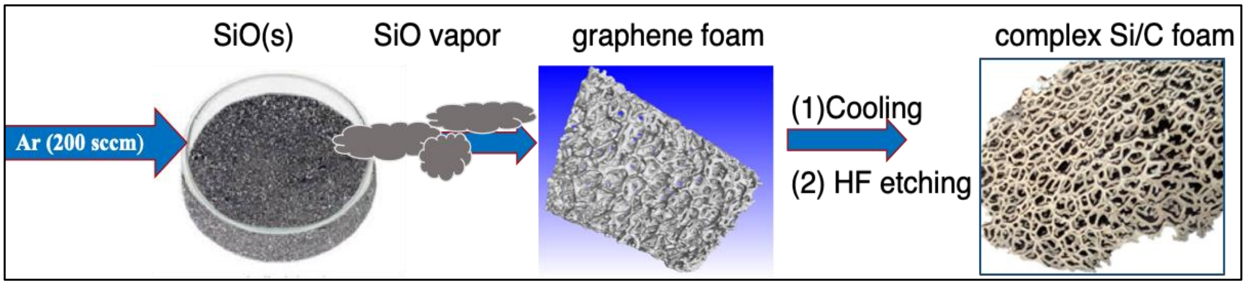

Figure S2. A schematic of the CVD synthesis of the foam.

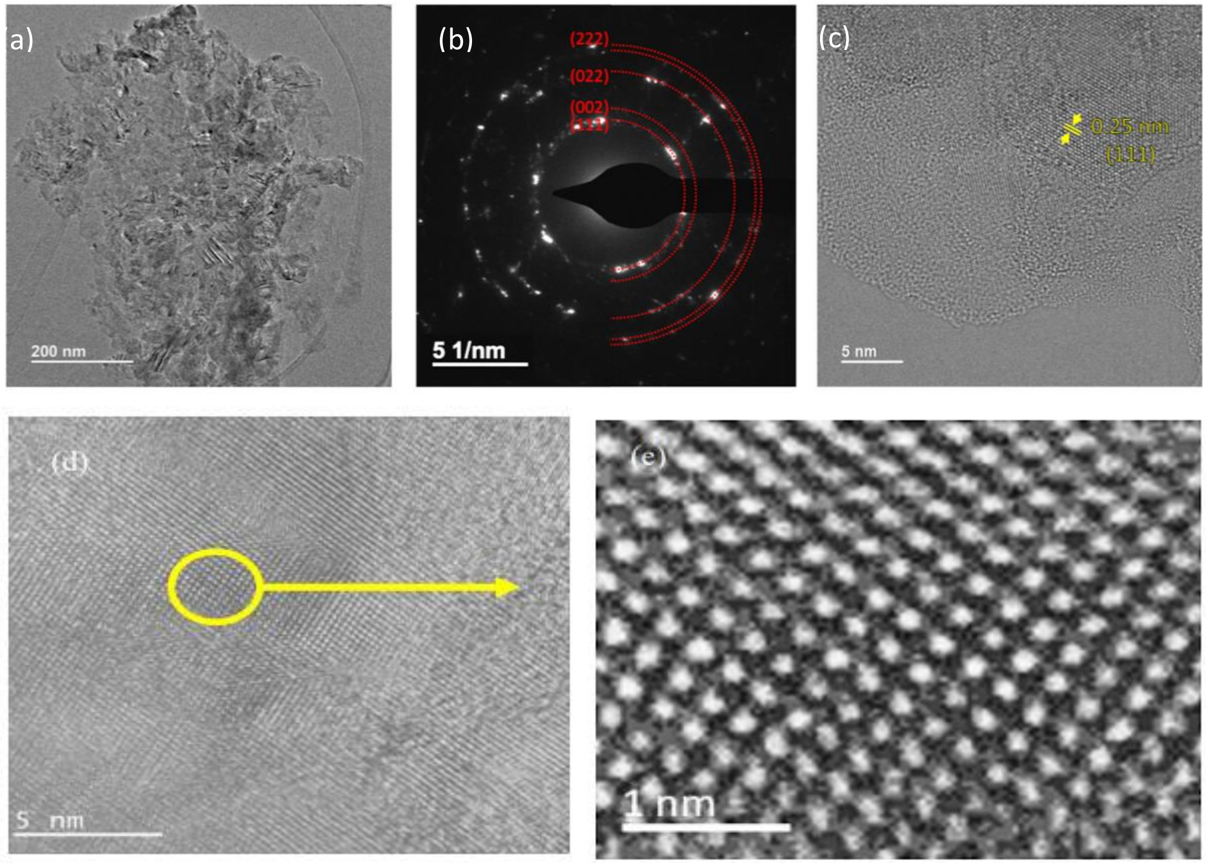

Figure S3. (a) TEM image and (b) indexed selected area electron diffraction pattern from a polycrystalline portion of the sample. (c-e) TEM images from different samples showing local crystalline order.

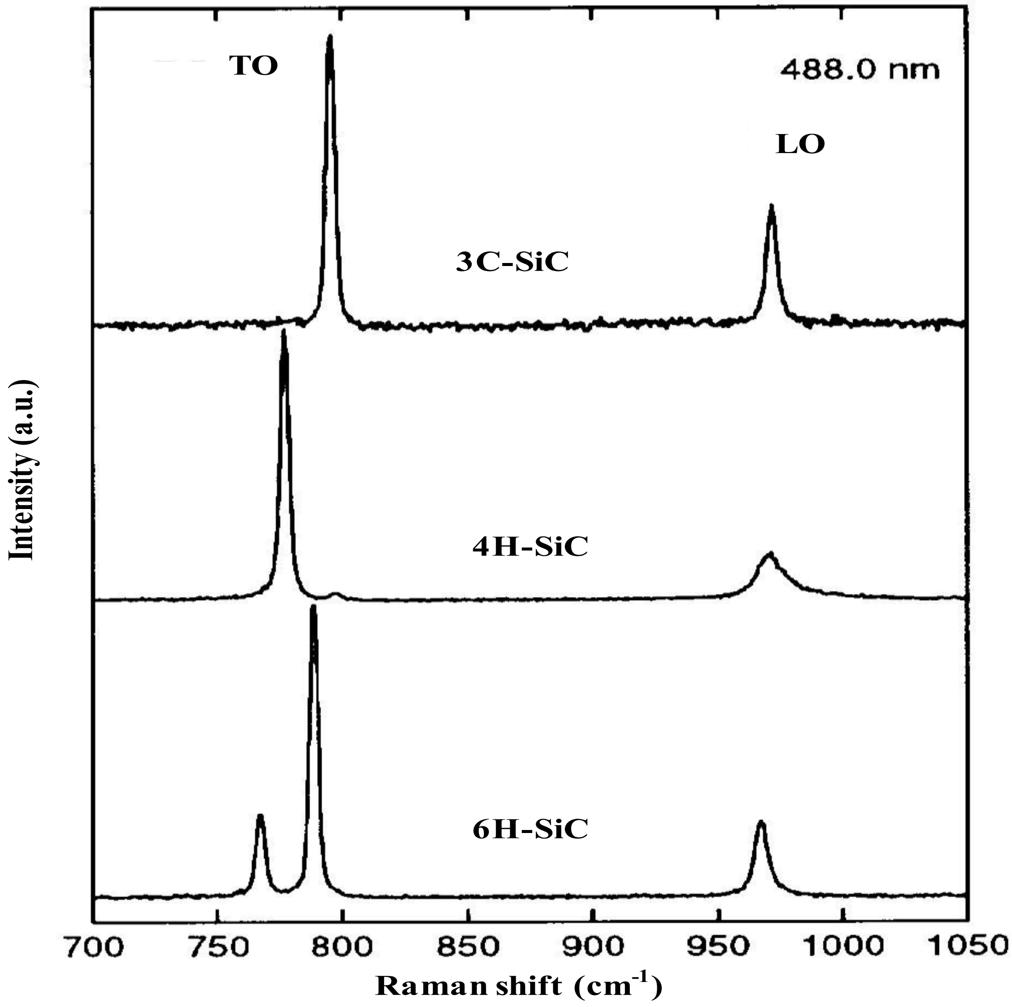

Figure S4. Raman spectra of different polytypes of SiC[1]. Reproduced with permission from Elsevier. Copyright 2015. Published by Elsevier Ltd.

Reference:

(1) Cantarero, A. Raman Scattering Applied to Materials Science. *Procedia Mater. Sci.* **2015**, *9*, 113–122.
